# Effect of leukoreduction in preventing febrile transfusion reactions

**DOI:** 10.6026/973206300212772

**Published:** 2025-08-31

**Authors:** Keshav R. Kulkarni

**Affiliations:** 1 Department of Pathology, S. Nijalingappa Medical College & H.S.K. Hospital & Research Centre, Nava Nagar, Bagalkote, Karnataka, India

**Keywords:** Leukoreduction, ionfebrile non-hemolytic transfusion reactions, PRBC, TACE 2 extractor, terumo penpol, blood safety, automated blood component separator

## Abstract

Febrile non-hemolytic transfusion reactions (FNHTRs) remain one of the most common adverse events associated with packed red blood cell (PRBC) transfusions. Therefore, it is of interest to compare the efficacy of leukoreduction in the prevention of febrile transfusion reactions in PRBC-transfused patients using the TACE 2 automatic component extractor and Terumo Penpol blood bags. 600 PRBC transfusions were included in the analysis, of which 300 were leukoreduced and 300 were not leukoreduced. The standard operating procedures for leukoreduction were adopted with the help of the TACE 2 device and the rate of FNHTRs was noted and compared between the two groups. Leukoreduction greatly minimized the occurrence of febrile non-hemolytic transfusion reactions. Its application with automated extraction systems such as TACE 2 and quality blood bags like those of Terumo Penpol provided safe, effective and repeatable results.

## Background:

Blood transfusion is a pillar of contemporary medicine, a key component of anemia, trauma, malignancy, surgical and chronic disease management. Although transfusion therapy has become safer and more routine, it does not exist without risks [1]. One of the most common adverse events is the febrile non-hemolytic transfusion reaction (FNHTR), a clinical syndrome defined by an unexplained increase in body temperature of ≥1°C (or 1.8°F) during or within four hours after transfusion and usually associated with chills, rigors, headache, or malaise [2, 3]. While usually non-life-threatening, FNHTRs may cause considerable patient distress, disruption of needed transfusions, added healthcare expenses and, in some instances, unwarranted diagnostic evaluation for fever [4]. FNHTRs are mainly due to the collection of leukocytes and their byproduct cytokines in the transfused blood product. Upon storage, WBCs from packed red cells and platelets inside blood bags go through apoptosis and necrosis with the release of pro-inflammatory cytokines, which include interleukin-1 (IL-1), tumor necrosis factor-alpha (TNF-α) and interleukin-6 (IL-6) [5, 6]. FNHTR's pathogenesis involves these mediators and renders leukoreduction, WBC removal from transfused blood, an efficient prophylaxis strategy. There are several ways of leukoreduction, which include pre-storage filtration, bedside filtration and apheresis. Among them, pre-storage leukoreduction has been demonstrated to be most effective as it removes leukocytes before significant cytokine accumulation can occur [7, 8]. This not only reduces the incidence of FNHTRs but also minimizes the risk of alloimmunization, transfusion-transmitted infections and immunomodulatory effects that can increase susceptibility to postoperative infections or cancer recurrence. In the Indian context, leukoreduction is not yet uniformly practiced due to resource constraints, equipment availability and cost considerations [9]. However, the increasing burden of transfusion-dependent patients and awareness about transfusion safety make an urgent need for evaluating and advocating evidence-based strategies such as leukoreduction. The Tace 2 leukoreduction device, when used with integral filter Terumo Penpol blood bags, presents a viable and standardized approach to obtaining high-quality pre-storage leukoreduction that can be incorporated into current transfusion practice [10]. Therefore, it of interest to assess the clinical efficacy of leukoreduction with the Tace 2 machine and Terumo Penpol blood bags in the prevention of FNHTRs in patients undergoing packed red blood cell (PRBC) transfusions in India.

## Materials and Methods:

## Study design and setting:

The research was planned as a prospective observational study over 1 year June 2024 to June 2025, in the Department of Pathology at S. Nijalingappa Medical College & H.S.K. Hospital & Research Centre, Nava Nagar, Bagalkote. The objective was to assess the efficacy of leukoreduction in the prevention of febrile non-hemolytic transfusion reactions (FNHTRs) among patients undergoing packed red blood cell (PRBC) transfusions. The study environment consisted of a specific blood component separation area with TACE 2 automatic component extractor and Terumo Penpol integral filter blood bags for preparation of leukoreduced PRBCs.

## Selection criteria:

All age groups and both genders of patients who needed PRBC transfusion during the study period were eligible. The inclusion criteria included patients receiving either leukoreduced or standard (non-leukoreduced) PRBC units with no history of febrile illness or active infection at the time of transfusion. Patients with known hemolytic reactions, transfusion-related acute lung injury (TRALI), or confirmed sepsis were excluded to prevent confounding causes of post-transfusion fever. Permission was received from all patients or their guardians before enrolment.

## Sample size calculation:

Under the assumption of a baseline incidence of FNHTR of 3% for non-leukoreduced units and a lower rate of 0.5% for leukoreduced units, with 5% significance level and 80% power (two-tailed), the minimum required sample size was estimated to be 250 patients per group. As a precaution to cover for possible dropouts or missing data, a total of 600 transfusion episodes (300 leukoreduced, 300 non-leukoreduced) were entered into the final analysis.

## Data sources and variables:

Leukoreduced PRBCs were manufactured following institutional SOPs with the use of the TACE 2 automatic component extractor. The process involved was initiation of the air compressor, system self-test, program selection (Program 1 for TAB first separation) and the correct routing of tubes using clamps and flow regulators. PRBCs were separated under aseptic closed conditions and leukoreduction was done with integrated in-line filters on Terumo Penpol bags. For every leukoreduced unit, rigorous adherence to operating instructions was adhered to, including protocol for centrifugation, placement of tubes and positioning of primary bags to guarantee uniform separation and filtration. Data were gathered through a standardized pro forma that captured patient demographics, indication for transfusion, type of PRBC unit transfused (leukoreduced or standard) and the development of FNHTR within four hours of the transfusion. FNHTR was characterized as an increase in temperature ≥ 1°C from baseline during or shortly following transfusion, not due to any other clinical etiology and sometimes associated with chills, rigors, or malaise.

## Statistical analysis:

Data were entered and analyzed using SPSS version 25.0 (IBM Corp., Armonk, NY). Categorical data were reported as frequencies and percentages and continuous data were expressed as means ± standard deviation. The Chi-square test or Fisher's exact test was used to compare the incidence of FNHTRs in leukoreduced and non-leukoreduced groups. A p-value <0.05 was used to determine statistical significance. Subgroup analysis was performed by age, gender and patient number of transfusions to examine any modifying effects on the rate of FNHTRs.

## Results:

600 transfusion episodes were studied over the study period, comprising 300 leukoreduced and 300 non-leukoreduced PRBC transfusions. The two groups were demographically similar, assisting in ensuring the validity of comparisons of outcomes. Table 1 (see PDF) presents the baseline characteristics of the study population. The patients' mean age in the leukoreduced group was 42.3 ± 16.7 years, whereas it was 41.5 ± 15.9 years in the non-leukoreduced group. The gender was also comparable in the two groups, with male gender constituting 54.3% and 56.0% respectively. The primary indication for blood transfusion across the two populations was anemia of chronic disease, followed by surgical bleeding and anemia secondary to malignancy. The rate of febrile non-hemolytic transfusion reactions (FNHTRs) was much lower in the leukoreduced group than in the non-leukoreduced group. As can be seen from Table 2 (see PDF), only 2 FNHTRs were reported among the 300 leukoreduced transfusions (0.67%), while 19 FNHTRs were reported in the non-leukoreduced group (6.3%). This difference was significant at p < 0.001, which suggests that leukoreduction was very effective in decreasing transfusion-related febrile reactions. Clinical presentation and timing of the reactions are summarized in Table 3 (see PDF). For both groups, FNHTRs generally occurred during the first two hours after the transfusion had begun. Within the leukoreduced group, two patients had mild fever, with one case involving chills. For the non-leukoreduced group, all 19 patients had fever and the majority had chills and rigors as well. None of the FNHTRs needed invasive therapy or hospitalization. Subgroup analysis was also performed to assess the effect of the number of units of PRBC transfused on FNHTR incidence. Table 4 (see PDF) emphasizes that FNHTRs occurred more frequently in patients receiving two or more units in the non-leukoreduced group. In the recipients of a single unit, FNHTR was 3.6% in the non-leukoreduced group and 0.4% in the leukoreduced group. The rate was significantly greater (11.6%) in recipients who received two or more non-leukoreduced units than in the leukoreduced group, which accounted for only 0.8%. These observations highlight the aggregate immunological effect of leukocyte exposure and the preventive function of leukoreduction, particularly with multi-unit transfusions. In general, the results of the study revealed a decline in FNHTR incidence due to leukoreduced PRBC transfusions derived with the TACE 2 component extractor and Terumo Penpol blood bags. No severe adverse events or process challenges were observed while performing the leukoreduction process, further supporting the practice acceptability of incorporating this protocol as a routine procedure in transfusion units.

## Discussion:

This research evaluated the effectiveness of leukoreduction in the prevention of FNHTRs with the use of PRBC units that were processed with the TACE 2 automated component extractor and Terumo Penpol blood bags. The results firmly endorse the theory that leukoreduction significantly reduces the incidence of FNHTRs among transfusion recipients, redemonstrating the clinical advantage of this therapy [8, 11 and 12]. Our data demonstrated a significant decrease in FNHTRs among leukoreduced PRBC recipients versus standard (non-leukoreduced) recipients-0.67% vs 6.3%, respectively. This notable decrease is consistent with other studies that have pointed out residual leukocytes as major contributors to FNHTRs via cytokine accumulation and immune sensitization [3, 10]. The reduced frequency of reactions in our leukoreduced group favors the function of white cell filtration or buffy coat removal in reducing this pathophysiological cascade [10]. Moreover, subgroup analysis offered additional information regarding the cumulative risk of FNHTRs with multi-unit transfusions. In the non-leukoreduced arm, patients receiving two or more units had a disproportionately increased rate of FNHTR (11.6%) versus those receiving one unit (3.6%). This trend highlights the enhanced immunological load presented by successive exposures to leukocytes and underlines the need for leukoreduction in multi-unit transfusion settings [13, 14]. Employment of TACE 2 and Terumo Penpol blood packs enabled standardized and effective leukoreduction without any functional failures or procedural complications noted. The inbuilt SOPs ensured uniformity of component processing and reduced variability of the leukocyte depletion step, making the results more robust. Importantly, despite automation, residual FNHTRs in a minor number of leukoreduced components revealed that though leukoreduction is useful, it does not eliminate the risk entirely [15]. These results have clinical implications for transfusion regimens, especially in high-volume institutions or in those patients frequently transfused, such as oncology, surgical, or chronic illness populations. Instituting leukoreduction can markedly decrease reactions, enhance patient compliance and comfort and decrease post-reaction assessment or interventions [16, 17]. Universal leukoreduction programs have also been linked with other advantages, such as decreased mortality and antibiotic use in high-risk patient populations.

## Strengths and limitations:

A key strength of this research is the application of an automated and standardized method of leukoreduction by using the TACE 2 extractor, which maximized reproducibility and reduced operator variation. The sample size was adequate and the provision of a control non-leukoreduced group provided maximum opportunities to assess effect size. The study's real-world setting enhances the external validity and generalizability of the results to everyday clinical practice. But the study was not without limitations. It was at a single institution, which might restrict the external validity of the findings. FNHTRs were diagnosed clinically without confirmation by laboratory tests (e.g., cytokine levels or leukocyte count in the final product) and this might have introduced observer bias. Moreover, patients were not followed up after the acute post-transfusion period, so delayed reactions to transfusion could not be evaluated. Finally, although FNHTRs were the focus of the current research, additional advantages of leukoreduction were not investigated.

## Conclusion:

Leukoreduction of PRBC units with the TACE 2 extractor and Penpol Terumo blood bags decreases the incidence of febrile non-hemolytic transfusion reaction significantly. The procedure proved to be efficient, standardized and practical even for day-to-day use within standard blood banking settings.
